# Minority Stress, Self-Awareness, and Coping Strategies during the COVID-19 Pandemic among Italian Transgender Young Adults

**DOI:** 10.3390/healthcare12020132

**Published:** 2024-01-06

**Authors:** Veronica Della Casa, Alessio Gubello, Anna Malmquist, Selene Mezzalira, Marina Bonato, Alessandra Simonelli, Michela Gatta, Marina Miscioscia

**Affiliations:** 1 Department of General Psychology, University of Padova, Via Venezia 12, 35131 Padua, Italy; veronica.dellacasa@studenti.unipd.it (V.D.C.); marina.bonato.1@studenti.unipd.it (M.B.); 2 DéFaSy, Faculty of Psychology, Educational Sciences and Language and Speech Therapy, Université Libre de Bruxelles, 1050 Brussels, Belgium; alessio.gubello@ulb.be; 3 Department of Behavioural Sciences and Learning, Linkoping University, Campus Valla, I-Huset, 3, 581 83 Linkoping, Sweden; anna.malmquist@liu.se; 4 School of Engineering, University of Basilicata, Via Nazario Sauro, 85, 85100 Potenza, Italy; selene.mezzalira@unibas.it; 5 Department of Developmental Psychology and Socialization, University of Padova, Via Venezia 8, 35131 Padua, Italy; alessandra.simonelli@unipd.it; 6 Department of Women’s and Children’s Health, University of Padova, Via Giustiniani 3, 35128 Padua, Italy; michela.gatta@unipd.it

**Keywords:** transgender, mental health, gender minority stress, LGBTQ+, COVID-19

## Abstract

Background: The security measures implemented in response to the COVID-19 emergency have caused complex consequences. The aim of the present study is to examine the repercussions of the pandemic on individuals belonging to gender identity minority groups, who have experienced heightened levels of stress in comparison to the general population. Methods: Online interviews with 12 transgender participants who resided in Italy during the pandemic were conducted and subsequently analyzed following the thematic analysis methodology. Results: The majority of the participants reported an increase in stress levels primarily attributed to the lack of acceptance and support within their familial environments, obstacles encountered in accessing specialized healthcare services, and a lack of support from the broader LGBTQ+ community. Despite these challenges, several participants developed effective coping strategies and a subset of them also benefited from multiple resilience factors, including familial support and assistance from mental health professionals. Conclusions: The outcomes of the present study indicate that the COVID-19 pandemic, while fostering certain protective factors within this population, has also given rise to new and critical mental health concerns. These findings hold significant implications for professionals working with transgender populations, highlighting the necessity of addressing these emerging mental health issues.

## 1. Introduction

In an effort to mitigate the spread of the SARS-CoV-2 virus, the Italian government implemented a series of restrictive measures targeting the entire population from March 2020 to March 2022 [1,2]. These measures involved alternating periods of stringent lockdowns, which entailed restrictions on leaving one’s residence and traveling outside one’s municipality except for essential purposes, and interludes during which these restrictions were eased. Consequently, educational institutions, including schools and universities, sports facilities, and non-essential retail stores were compelled to suspend their activities and evening curfew regulations were instituted. These stringent measures had a profound impact on the mental well-being of the Italian population, as a result of related psychosocial phenomena, such as prolonged confinement, heightened uncertainty concerning the pandemic’s trajectory, the absence of robust social support networks, the proliferation of misinformation via social media, and the associated negative affectivity [3,4], in conjunction with the economic crisis [5,6]. On a national scale, during the initial wave of the pandemic, spanning from April to May 2020, there was a twofold increase in the prevalence of psychopathological symptoms, affecting more than one third of the general adult population [7]. In addition to elevated rates of post-traumatic stress disorder (PTSD) associated symptomatology, COVID-19 was related to depression, anxiety, insomnia, and stress [8,9]. Notably, the severity of these symptoms increased over time, becoming more pronounced in the last weeks of the lockdown, in comparison to the initial phases [4].

Marginalized groups, such as transgender individuals, experienced disproportionately adverse effects during the pandemic, driven by the heightened vulnerability to psychological distress due to the gender minority stress they experience daily [10,11,12]. The “minority stress theory”, developed by Meyer [13,14], posits that individuals belonging to social minority groups are exposed to specific, additional stressors resulting from living with a stigmatized identity that makes them more vulnerable to developing mental health problems. Accordingly, minority stressors are organized on a distal–proximal axis: whereas distal stressors are due to external factors that marginalize or threaten the safety and security of minority groups (e.g., discrimination, harassment, etc.), while proximal stressors refer instead to the subjective experience of minority groups facing a world experienced as oppressing, stigmatizing, and unsafe (e.g., internalized stigma, expectation of rejection, etc.). The minority stress theory also recognizes the fundamental role played by resilience factors in buffering the negative effect that minority stressors have on minority groups’ health outcomes [15]. In order to address the specific experience of transgender and gender diverse (TGD) individuals, the minority stress theory has been then expanded by Testa et al. [16] into the “gender minority stress and resilience theory”, which recognizes the peculiarity of the minority stressors (e.g., non-affirmation, transnegativity, etc.) and resilience factors (e.g., self-definition and medical or social transition) affecting this specific population.

Studies conducted on the Italian transgender population during the COVID-19 pandemic unveiled elevated levels of depression, hostility, anxiety, and discouragement about the future, in conjunction with discernible negative changes in psychological well-being, evident when comparing the pre-pandemic and pandemic emergency periods [17,18]. Globally, a multitude of studies documented psychological distress within the transgender community, due to the high prevalence of discontinuation or delays in hormone therapy administration and/or gender affirmation surgery during the pandemic [19,20,21], as well as reduced access to specialized support and services for LGBTQ+ individuals [19,21], including the closure of LGBTQ+ associations and support networks. Despite the documented impact of this health emergency on the overall quality of the transgender individual’s well-being, there is a lack of qualitative investigations that explore their emotional experiences, challenges, and available resources, particularly in the context of minority stress. Consequently, the principal objective of this study is to qualitatively delve into the challenges and opportunities encountered by Italian transgender individuals during the COVID-19 pandemic.

## 2. Materials and Methods

The study engaged 12 participants self-identifying as transgender, whose ages ranged from 18 to 43 years (M = 26.41; SD = 10.50). Ten of these participants identified as transgender men, of which nine were assigned female at birth (AFAB), and one was assigned intersex at birth. The remaining two participants identified as transgender women and were assigned male at birth (AMAB). Further sociodemographic characteristics are described in Table 1.

This project has been approved by the Ethical Committee of Psychological Research, Area 17, at the University of Padova (n. 4257/2021). The criteria for participant inclusion stipulated that individuals should be 18 years of age or older, self-identify as a transgender man or transgender woman, and have resided predominantly in Italy during the pandemic period. Participants were recruited from the cohort that participated in the study “Social Network Support and Psychological Well-Being among young LGBTQ+ persons during the COVID-19 pandemic—Follow-up Phase through interviews” [22]. Survey respondents were recruited through a variety of means, including contacts with LGBTQ+ associations, referrals via word of mouth, and social media (Facebook and Instagram). At the end of the survey, participants were invited to express their willingness to participate in follow-up interviews, and those who consented were subsequently contacted. Prior to the beginning of the interviews, an informed consent form was forwarded via email and signed by each participant. All interviews were conducted via the Zoom platform from 13 April 2021, through 28 February 2022, with durations spanning from 30 to 90 min. All of the interviews were recorded and were conducted utilizing a semi-structured interview guide comprising 13 questions, developed by Anna Malmquist et al. [23,24]. The primary objective of these questions was to explore the participants’ experience of their health and social situation before and during the COVID-19 pandemic. This encompassed an examination of the influence of containment measures on their daily lives, mental health, and experiences associated with gender minority stress. Through the collected interviews, information power [25] was reached.

Subsequent to the interviews, the complete audio recordings were transcribed verbatim, and the resulting transcripts were subject to review and analysis, employing the thematic analysis methodology [26,27]. Each interview underwent independent coding by two researchers, with each code representing the participant’s reflections on their personal experiences or feelings. These codes were then aggregated into sub-themes, and then into general themes. Following the delineation of these general themes, a summary paragraph was drafted for each of them. Presented here are the identified themes associated with the significance of restrictions for transgender individuals and how stigma and discrimination impacted the lives of the participants during the pandemic.

## 3. Results

The majority of the study participants reported experiencing mental health consequences that paralleled those observed within the broader Italian population. These outcomes were primarily attributed to the persistent denial reality and the lack of social interactions under the pandemic restrictions. Among these consequences were heightened levels of general stress, increased irritability and nervousness, augmented physical and mental fatigue during the execution of daily activities, a sense of being static and oppressed, feelings of uncertainty and fragility, and a lack of future perspectives. A subset of individuals experiences severe mental health disorders or observed an exacerbation of pre-existing conditions they had experienced years earlier, such as generalized anxiety disorders, panic attacks, intense fear of dying, insomnia, or difficulties in navigating social settings. Within this context, the heightened experiences including the physical, social, and emotional dimensions ascribed to transgender identity were significantly accentuated. This study has identified two major themes that address this phenomenon, namely, “Lockdown-related advantages and disadvantages for transgender people” and “Minority stress, social stigma, and discrimination during the pandemic”.

### 3.1. Lockdown-Related Advantages and Disadvantages for Transgender People

#### 3.1.1. “I Discovered This Aspect of My Identity”: Exploring and Expressing Gender

The lockdown period was often regarded as a respite from the pressures of societal expectations, providing a unique opportunity to regain a sense of balance and renewed motivation. In contrast to the frenetic pace of daily life, the pandemic-induced restrictions granted individuals the time to delve into deeper introspection regarding who they are. For some participants, this period served for the exploration or re-exploration of their gender identity.

*I have had positive repercussions. Because I had the time to take those spaces to understand who I was and what I wanted. So definitely on the positive side and definitely if [the pandemic] hadn’t been I wouldn’t be at this point [in the gender affirmation path].*

Nicola, 19, intersex, transgender man

The period of isolation at home prompted individuals to engage in self-reflection and explore gender expressions in a relaxed and playful manner. For instance, Giorgio, a transgender man, started to elaborate on his more feminine aspects by reintegrating clothes that he had worn prior to beginning his gender affirmation path. In the absence of the enforced isolation, he would not have felt at liberty to do so due to concerns about potential invalidation as a man by others. This experience led to a critical reevaluation of his concept of masculinity and the rejection of certain toxic elements that had been assimilated under social pressures. However, the protracted confinement at home engendered strenuous effects as well. The inability to present oneself publicly and have external validation of one’s gender identity led some participants into a profound sense of apathy or compelled them to prematurely disclose their gender identity with a forced coming-out to seek family assistance and recognition, since it was not possible to receive such validation by someone else.

#### 3.1.2. “My Doctors Have Disappeared”: When Pandemic Set Transition on Hold

Some participants who were actively engaged in the process of physically affirming their gender identity faced significant challenges as their transition was either placed on hold or significantly delayed. The restrictions on travel and limitations and delays in specific healthcare services stemming from the pandemic had an adverse impact on their gender affirmation pathway. This situation was perceived as an obstacle to their self-determination and freedom of expression, resulting in the discontinuation of treatments and regression in ongoing changes. The postponement of gender affirmation treatments for several months led to a range of issues, including depression, intense anger, hair loss, loss of appetite, significant mood swings, setbacks in their physical changes, a pervasive sense of emptiness and darkness. Another distressing factor during this period was the absence of support from the professionals who were guiding the participants through their gender affirmation process, at times seeming to vanish without a trace: “disappeared into thin air”. Financial constraints stemming from job loss or other pandemic-related complications also impeded and slowed some individuals on their path to gender affirmation.

### 3.2. Minority Stress, Social Stigma, and Discrimination during the Pandemic

#### 3.2.1. “So Much Nastiness in Certain Groups”: New Minority Stress Contexts

Some participants reported experiencing micro-aggressions from healthcare or administration services during the handling of green passes or vaccination certificates, which were required to access public facilities. For people who had not yet officially changed their registry information and presented with documents that had not been rectified, this situation resulted in incidents of misgendering and the use of dead names to the extent that some participants refrained from going out due to the stress caused by these experiences.

*Unfortunately, I had with the various offices not very pleasant situations. (…) sometimes someone thought that I had stolen them [documents] or in short that they belonged to another person. (…) You are in that situation where you have to, in some way, give credence to what you say, however, you don’t want to expose yourself and it’s definitely a stressful situation.*

Pietro, 22, AFAB, transgender man

Emerging evidence also sheds light on the phenomenon of online discrimination. The increased amount of time spent on social media and the accumulation of stress and frustration during the lockdown were exacerbating factors for the occurrence of hate speech. This phenomenon was not limited to the general population, but extended to the LGBTQ+ community itself. Insults and discrimination on social media, targeting LGBTQ+ individuals, were widely reported. Furthermore, there was a notable lack of representation for certain subgroups within the LGBTQ+ community, such as disabled LGBTQ+ individuals or those with diverse relational or romantic orientations. Conversely, there was a notable increase in the presence and attention given to LGBTQ+ individuals on social media platforms, with a specific focus on transgender rights. This allowed some participants to come out or to engage in activism on the internet or within LGBTQ+ associations. One of the challenges faced by LGBTQ+ individuals during the pandemic was the absence of safe spaces where they could express themselves without fear of discrimination or attacks. Although many LGBTQ+ associations continued to hold online meetings, these did not entirely replicate the value of in-person interactions. Some participants occasionally violated restrictive rules to meet fellow members of LGBTQ+ associations, considering them as the only sources of support and security during this period. For them, the importance of finding safety and support outweighed the need to adhere strictly to pandemic restrictions. The Italian LGBTQ+ community also engaged in a national discourse regarding exemptions from government-mandated isolation, particularly concerning the concept of “stable affection”. Initially, the entire community stood united against a binary and heteronormative interpretation of “stable affection”. However, when it became evident that same-sex couples were also encompassed within this definition, divisions emerged within the LGBTQ+ community. These divisions centered around concerns that the definition excluded individuals who were not engaged in romantic relationships, ultimately resulting in participants feeling “alienated from the (LGBTQ+) community during the whole pandemic”. In terms of specific strategies employed to address stigma, a heightened inclination toward confrontation and open dialogue had emerged in order to denounce discriminatory situations and educate on mutual respect. For instance, in cases of misgendering, some participants expressed confidence in educating others about the proper use of pronouns and nouns of choice.

#### 3.2.2. “No One Was Looking at Me”: Feeling Safe under the Mask

Participants reported ambivalent attitudes about home isolation and mask usage. For some, these measures served as protective mechanisms toward exposure to external gaze and judgment, thereby reducing perceptions of minority stress and gender dysphoria. Additionally, some transgender women found that extended periods at home, without social events, relieved them of the social pressure to conform to cisgender norms in terms of appearance. They felt less compelled to undergo makeup routines or dress in ways to ‘pass’ as cisgender individuals, which positively impacted their mental well-being and reduced the fear of discrimination. For instance, Francesca found a sense of security in wearing a mask:

*I always have anxiety when I don’t have the mask on. I kind of experience these situations related not to the lockdown, but to the transition and the fact that I lived everything in lockdown, that is, even just discovering myself. I get hallucinating anxiety walking down the street without having the mask protecting me.*

Francesca, 33, AMAB, transgender woman

However, for other participants, wearing masks and enduring social isolation was a risk factor. These measures prevented them from displaying the physical changes resulting from hormone therapy, such as facial hair growth (beard or mustache), and from receiving social validation. In some cases, the use of masks led to participants being misgendered by others, resulting in feelings of invalidation. For example, Luca, a 23-year-old transgender man, reported:

*I started the medicalized transition at the end of 2019, so I started to see the changes right around the time I had to put the mask on; therefore, I couldn’t see nor could I show, I didn’t feel free to say ‘okay now this is me, because now I want others to see me’. There was this need and instead I couldn’t do it, and that really brought me a discomfort.*

Luca, 23, AFAB, transgender man

## 4. Discussion

These findings serve as an initial exploration, offering a foundation for a broader consideration of the experiences of individuals within the transgender community and how the pandemic, along with associated restrictive measures, has influenced their lives. Firstly, the majority of participants expressed a complex spectrum of feelings concerning the pandemic situation. While social isolation significantly impacted their emotional and personal lives, it also facilitated meaningful introspection and prompted significant life changes. More precisely, for some individuals, isolation and the consequent absence of stigma and social pressures provided an opportunity for the exploration of their gender identity and/or expression. This highlights the role of societal constraints in inhibiting the initiation of one’s social affirmation of gender identity.

This observation finds support in reflections made by some participants, highlighting the advantages of social isolation in terms of reduced perceptions of discrimination and minority stress during the initial stages of the social affirmation path and hormone therapy. It is worth noting that these early phases of the gender identity affirmation journey are often the most challenging, as transgender individuals often experience heightened vulnerability [28]. Therefore, these findings offer valuable insights into potential coping strategies that could be implemented during such a sensitive period, even though avoidance mechanisms may provide short-term relief, they may also contribute to a long-term increase in experienced minority stress [29,30].

Conversely, the social aspect assumes critical significance at a later stage of one’s gender affirmation path, when physical changes become noticeable and are perceived as integral to one’s identity and representative of one’s gender identity. The distress stemming from a lack of social validation for these physical changes and the inability to be recognized in one’s own gender due to the concealment of facial features by face masks resulted in the impossibility of self-determination. This factor negatively impacted the mental health of transgender individuals. Participants repeatedly expressed ambivalence toward various restrictive measures, including the use of face masks, which were perceived both as a potential threat as well as a source of protection from judgmental gazes or gender dysphoria concerning facial appearance.

Significant adverse consequences were observed among transgender individuals following a medically guided gender identity affirmation path. Delays in accessing hormone treatment, reduced specialist visits, and forced interruptions to hormone therapy had a profound impact on the mental well-being of some participants. Additionally, the inability to access these vital pathways privately due to reduced or nonexistent income reaffirmed concerns raised in previous studies [19,20,21,22,23]. Furthermore, the emotional vulnerability of participants undergoing hormone treatment was frequently overlooked by healthcare professionals. This amplified the already challenging experiences stemming from lockdowns and social restrictions, significantly impacting the transgender community [17,18].

These challenges were further exacerbated by discrimination against the LGBTQ+ community, particularly in online spaces, and the absence of secure environments for LGBTQ+ gathering and activism. These unmet needs resulted in some breaches of social distancing norms, highlighting the fundamental significance of these spaces for gender minorities [31]. Another crucial source of support significantly influencing the well-being of individuals was psychological assistance. Thanks to this support, many participants could effectively reframe and address the challenges arising from the pandemic-related restrictions. The discontinuation of this psychological support was deeply burdensome, especially for a population at heightened risk of experiencing psychological and emotional difficulties compared to the general Italian population [32,33,34].

A final and noteworthy finding was represented by the influence of the national context on the well-being of the participants. During the initial stages of the pandemic, characterized by stringent social isolation, perceptions of stigma and discrimination were relatively subdued. However, as the pandemic progressed through its second and third waves, two divisive situations emerged in the Italian national landscape. The first was the approval of legislation addressing homo-lesbo-bi-trans-phobia, while the second was the implementation of green passes attesting to COVID-19 vaccination. Both events, although at first glance appearing dissimilar, brought the nation’s attention to the transgender community. The first because it advocated for the right to be free from discrimination, while the second because it involved a highly discriminatory measure affecting transgender individuals who had not rectified their identification documents. At the same time, in many countries, the protests of the “Black Lives Matter” movement took place against every form of discrimination, shedding light on several minority groups affected by systematic discrimination. Numerous participants worldwide joined the BLM movement protesting for respect and non-discrimination, and its resonance played an important role in creating awareness about human rights; nevertheless, its echo seemed not to affect the Italian context, where public debate and political discussion were centered on the rejection of the bill against homo-lesbo-bi-trans-phobia (DDL 4 November 2020; see [22]). Consequently, during this period, participants reported a significant increase in attacks targeting the transgender community, along with elevated levels of social stigma and minority stress. These challenges were further exacerbated by difficulties in finding safe and supportive environments [31], ultimately leading to the onset and/or worsening of anxiety and depressive symptoms [35].

The results obtained should be interpreted in the context of certain limitations. Firstly, the necessity to conduct interviews entirely online due to the COVID-19 restrictions occasionally resulted in issues related to internet connectivity, background noise, distractions, and the absence of non-verbal cues. Furthermore, the role played by the researchers and the flexibility employed in qualitative analysis, while invaluable for understanding the phenomenon, may pose challenges in terms of replicating the obtained outcomes. However, this study also has noteworthy strengths. To begin with, the phases involving participant recruitment and data collection spanned the entire duration of the pandemic, facilitating the accumulation of representative accounts of the phenomenon as a whole. Moreover, the employment of semi-structured interviews as a methodology fostered trust and facilitated participants in offering comprehensive narratives of their experiences, allowing them the freedom to elaborate and provide in-depth accounts of their lived experiences. For future research, a longitudinal approach should be considered, examining how individuals’ experiences and attitudes have evolved over time and whether further implications for mental health have emerged in the post-pandemic landscape.

## 5. Conclusions

Based on the findings that have emerged from our research, some final considerations are warranted. Firstly, this study has provided a comprehensive overview of the conditions characterizing the Italian transgender population during the COVID-19 pandemic. In addition to the common challenges experienced by the broader population, such as the discomfort associated with the imposition of restrictions, we have identified critical issues specific to the transgender community. These challenges encompass the absence of external support systems, obstacles in accessing healthcare services, and the identification of restrictions as tools for self-protection against discrimination. These challenges compound an already burdensome condition for these individuals, who are subjected daily to individual and societal challenges related to their gender minority status. However, the presence of protective factors, independently developed by participants during the pandemic, holds promise, suggesting a wealth of resources that can serve as a defense against social stigma, contributing to protecting this community.

The results presented in this study contribute to a better comprehension of the specific stressors that the transgender population in Italy encountered during the COVID-19 pandemic and some strategies participants put into practice to preserve themselves and their mental health. In this vein, some of the emerged themes explicitly refer to the Italian transgender community and difficulties related to living in this country, such as the forementioned public debate on the bill against homo-lesbo-bi-trans-phobia or the implementation of the green pass policy. On the other hand, most narratives appear comparable with what the transgender community experienced in other countries during the COVID-19 pandemic, in particular the worsened mental health conditions, the lower perception of social support, the higher levels of hate against transgender people and the absence of safe spaces for the community [23,24]. In this sense, this study highlights specific and common stressors that the transgender community met in various countries and different settings during COVID-19 emergency, providing a broader frame for professionals and policies.

The lived experience during the period of restrictions considerably affected individuals’ psychological and overall health. In these situations, the most vulnerable population faces higher challenges with a higher cost regarding well-being outcomes. The LGBTQI+ population is still considered vulnerable, particularly transgender people who, in different parts of the world, including Italy, experience numerous stressors and structural discrimination [24]. Anti-discrimination policies need to be substantially developed in our country. Transgender people who have not had access to rectification ID documents have been exposed to continuous requests for green pass identification. Incongruent data concerning physical appearance and identity have exposed some people to discriminatory acts and bullying, as well as the suffering of forced coming out and disrespect for privacy. Institutional awareness needs to be ensured concerning the limitations of present welfare policies for transgender people so that new policies can be developed that take into account specific needs such as those found in this study.

## Figures and Tables

**Table 1:** Sociodemographic Characteristics of the Sample.

Variables	Sample (*n* = 12) *n* (%)
*Age* (*mean*)	26.41 (SD = 10.50)
*Assigned gender at birth*	
Male	9 (75%)
Female	2 (16.7%)
Intersex	1 (8.3%)
*Gender identity*	
Transgender man	10 (83.3%)
Transgender woman	2 (16.7%)
*Sexual orientation*	
Heterosexual	5 (41.3%)
Homosexual	1 (8.3%)
Asexual	1 (8.3%)
Queer	2 (16.7%)
Questioning	2 (16.7%)
No sexual orientation	1 (8.3%)
*Occupational status*	
Student	4 (33.3%)
Working student	3 (17.1%)
Employed	4 (38%)
Unemployed	1 (8.3%)
*Highest level of education*	
Second grade	9 (75%)
Bachelor degree	2 (16.7%)
University Master	1 (8.3%)
*Current relationship status*	
Partnered	6 (50%)
Single	6 (50%)

## Data Availability

The data presented in this study are available on reasonable request from the corresponding author.
